# Aberrant Functional Network of Small-World in Sudden Sensorineural Hearing Loss With Tinnitus

**DOI:** 10.3389/fnins.2022.898902

**Published:** 2022-05-19

**Authors:** Jin-Chao Hua, Xiao-Min Xu, Zhen-Gui Xu, Jin-Jing Xu, Jing-Hua Hu, Yuan Xue, Yuanqing Wu

**Affiliations:** ^1^Department of Otolaryngology, Nanjing Pukou Central Hospital, Pukou Branch Hospital of Jiangsu Province Hospital, Nanjing, China; ^2^Department of Radiology, Nanjing First Hospital, Nanjing Medical University, Nanjing, China; ^3^Department of Otolaryngology, Nanjing First Hospital, Nanjing Medical University, Nanjing, China

**Keywords:** sudden sensorineural hearing loss, tinnitus, small world, cortical connectivity, graph theoretical

## Abstract

Few researchers investigated the topological properties and relationships with cognitive deficits in sudden sensorineural hearing loss (SNHL) with tinnitus. To explore the topological characteristics of the brain connectome following SNHL from the global level and nodal level, we recruited 36 bilateral SNHL patients with tinnitus and 37 well-matched healthy controls. Every subject underwent pure tone audiometry tests, neuropsychological assessments, and MRI scanning. AAL atlas was employed to divide a brain into 90 cortical and subcortical regions of interest, then investigated the global and nodal properties of “small world” network in SNHL and control groups using a graph-theory analysis. The global characteristics include small worldness, cluster coefficient, characteristic path length, local efficiency, and global efficiency. Node properties include degree centrality, betweenness centrality, nodal efficiency, and nodal clustering coefficient. Interregional connectivity analysis was also computed among 90 nodes. We found that the SNHL group had significantly higher hearing thresholds and cognitive impairments, as well as disrupted internal connections among 90 nodes. SNHL group displayed lower AUC of cluster coefficient and path length lambda, but increased global efficiency. The opercular and triangular parts of the inferior frontal gyrus, rectus gyrus, parahippocampal gyrus, precuneus, and amygdala showed abnormal local features. Some of these connectome alterations were correlated with cognitive ability and the duration of SNHL. This study may prove potential imaging biomarkers and treatment targets for future studies.

## Introduction

According to World Health Organization, hearing loss affects over 5% of the world’s population and will be up to one in every ten people by 2050. Additionally, 1/3 of worldwide hearing loss patients had severe impairment [World Health Organization [WHO], 2021]. Sudden sensorineural hearing loss (SNHL) is one of otologic emergency, which is defined as hearing loss in three consecutive frequencies within 3 days (Li et al., 2022). More than 80% of SNHL patients have been found to be accompanied by tinnitus which often lasts for > 6 months (Diao et al., 2021). After treatment, some SNHL can recover from hearing loss, however, the symptom of tinnitus still exists intermittently or continuously (Qi et al., 2021). SNHL is not only affect the structure/function of inner ear (including cochlear hair cells, auditory nerve fibers, and membranous labyrinth) but also induces central system symptoms (such as cognitive deficits, depression, and anxiety) (Castaneda et al., 2019), leading to poor quality of life.

Some cross-sectional and longitudinal studies about congenital and aging deafness have demonstrated that hearing loss is associated with cognitive impairments and a high risk of incident dementia, since hearing ability is one of the important sensory determinants in normal and pathological cognitions (Choi et al., 2021; Lau et al., 2022). Previous research (Croll et al., 2020) has illustrated whether the hearing loss in midlife (19–78 years) could contribute to dementia, and the hazard ratio is 1.90 before age 60 or 1.15 at a later age. The generally accepted potential mechanism is that effortful sensory perception after hearing loss results in an increase in cognitive load and a decrease in cognitive reserves on a vulnerable brain (Wayne and Johnsrude, 2015). Compared with unilateral SNHL, bilateral SNHL showed a worse prognosis and developed into a long-term hearing loss (Akil et al., 2017).

Functional MRI based on blood oxygenation level-dependent is an emerging non-invasive tool and has been widely used to explore the impaired cognition in subjects with hearing loss and neuropsychiatric disorders (Schouten et al., 2016; Meijboom et al., 2017; Puschmann and Thiel, 2017; Zhang et al., 2018). However, only a few studies focused on the whole brain network organization. Graph theory technology uses a mathematical framework to characterize the human brain’s underlying architecture, as a large-scale network comprising a set of nodes (brain regions) and edges (connectivity strength) (Wilke et al., 2011; Medaglia, 2017). Different from dynamic functional connectivity (FC) and independent component analysis (ICA), graph theory is of a moderate to high reliability and defines the whole brain as a “small-world network” (Braun et al., 2012), which is originally from social, economic, and biological concepts (Strogatz, 2001). Compared with a random network, a small world has higher specialization and global integration between nodes (Achard et al., 2006). Both the healthy brain and disease brain have small-world properties, while the topologic alterations have been found in various disease brains, namely, Alzheimer’s disease (Stam et al., 2009), depression (Li et al., 2015), schizophrenia (Lynall et al., 2010), and multiple sclerosis (He et al., 2009). To the best of our knowledge, research on deafness using graph theory analysis are limited and most of them concentrated on unilateral SNHL or prelingual hearing loss (Kim et al., 2014; Xu et al., 2016).

Therefore, this study aimed to uncover the topologic properties of brains with bilateral SNHL with tinnitus. (1) We built the small-world network and investigated the difference between SNHL and healthy controls (HCs); (2) According to the AAL atlas (17), we chose 90 nodes to represent the whole brain and further explore the nodal features, plus interconnectivities between nodes following SNHL; (3) Finally, we assessed the relationship between changes in brain network and cognitive impairments.

## Materials and Methods

### Ethics Statement

This study was approved by the Research Ethics Committee of the Nanjing Medical University. All the procedures were in accordance with the Declaration of Helsinki (World Medical Association, 2013) and written informed consent was obtained from each enrollee at the beginning.

### Subjects

A total of 36 subjects with bilateral SNHL with tinnitus and 37 gender-, age- and education level matched healthy controls (HCs) were recruited from the local hospital and community for this study, all participants were right-handed and educated for at least 6 years (Table 1). The inclusion criteria of SNHL were as follows: (1) the level of hearing loss was > 30 dB in at least three contiguous frequencies; (2) duration < 1 month. The hearing threshold of HCs was <25 dB in the left and the right ear. The exclusion criteria were (1) conductive deafness, prelingual deafness, or presbycusis; (2) pulsatile tinnitus, Meniere’s disease, hyperacusis, otosclerosis; (3) inflammation of the external or middle ear; (4) severe lung, heart, liver and kidney diseases; (5) past history of head surgery, brain tumor, stroke, Parkinson’s disease, Alzheimer’s disease, drug or alcohol addiction; (6) suffered from major depression, anxiety or other psychiatric disorders; and (7) MRI contraindication.

**TABLE 1:** Demographic and clinical features of bilateral SNHL with tinnitus and HCs.

Characteristics	SNHL (*n* = 36)	Control (*n* = 37)	*p* value
Gender (male/female)	23/13	16/21	0.102
Age (years)	55.6 +9.7	52.6 +10.1	0.194
Education (years)	11.1 +3.1	13.1 +6.3	0.084
Duration (days)	7.7 +8.5	/	/
Hearing threshold-left ear (dB)	58.3 +20.9	15.2 +5.0	< 0.001
Hearing threshold-right ear (dB)	54.8 +19.8	17.5 +5.8	< 0.001
MMSE	29.6 +0.8	29.7 +0.6	0.564
SDMT	33.7 +12.2	42.7 +10.3	0.001
AVLT-first time	3.6 +1.5	4.6 +1.6	0.009
AVLT-second time	5.8 +1.8	6.6 +1.8	0.054
AVLT-third time	7.3 +1.8	7.5 +1.6	0.518
AVLT-5 min	5.8 +2.5	6.9 +1.3	0.024
AVLT-20 min	5.6 +2.5	6.6 +1.4	0.057

*Data are expressed as mean +SD.*

*SNHL, sensorineural hearing loss; HCs, healthy controls; MMSE, mini-mental state examination; SDMT, symbol digit modalities test; AVLT, auditory verbal learning test.*

The SNHL was diagnosed by a pure tone audiometry (PTA) test, which was conducted by a professional clinical audiologist from the E.N.T department of our hospital using a GSI-60 audiometer. The hearing thresholds at 0.25, 0.5, 1, 2, 4, and 8 kHz were all assessed in the present study.

Every subject underwent neuropsychological assessments before MRI scanning, including mini-mental state examination (MMSE), symbol digit modalities test (SDMT), auditory verbal learning test (AVLT), self-rating anxiety scale (SAS), and Hamilton depression scale (HAMD), which has been described in our previous study (Xu et al., 2019b).

Demographics and clinical data (such as PTA and neuropsychological tests) were compared by independent-sample *t*-test using SPSS software (version 21.0, Chicago). Categorical variables (e.g., gender) were compared using the Chi-square test. Significant *p*-value was set at < 0.05.

### MRI Data Acquisition

Subjects underwent resting-state scanning using 3.0 Tesla MRI with an 8-channel head coil (Ingenia, Philips Medical Systems, Netherlands). Participants were asked to lie quietly in the MRI scanner with their eyes closed, stay awake, and avoid thinking about special things. Earplugs plus earphones (Hearos Ultimate Softness Series, United States) were used to reduce scanner noise, and a head cushion was used to control head motion.

Structure images were collected by T1-weighted 3D turbo fast echo sequence to diagnose brain lesions: sections = 170, repetition time (TR) = 8.1 ms, echo time (TE) = 3.7 ms, section thickness = 1.0 mm, flip angle (FA) = 8°; field of view (FOV) = 256 mm × 256 mm, matrix = 256 × 256. Functional images were acquired using an echo planar imaging (EPI) sequence: slices = 36, TR = 2,000 ms, TE = 30 ms, thickness = 4.0 mm, FA = 90°, FOV = 240 mm × 240 mm, matrix = 64 × 64.

### Data Preprocessing

Our procedures for MRI data preprocessing were similar to prior studies (Xing et al., 2021; Xu et al., 2021). Data analysis was computed using GRETNA (Wang et al., 2015) toolbox^1^ based on MATLAB software^2^. Clinical DICOM data was firstly transferred to NIFTI data and the first 10 images were removed for signal equilibrium. The remaining 230 images were included in subsequent analysis, including slicing timing correction, realignment, normalization to EPI template, spatial smooth with a 6 mm full width at half maximum, and covariates regression using Friston-24 parameters (6 motion parameters, 6 temporal derivatives, and 12 corresponding squared items) (Yan et al., 2013). After that, linear drift and temporal filter (0.01–0.08 Hz) were conducted to eliminate the influence of physical noise. Subjects with a translational or rotational head motion of > 2.0 mm or 2.0° in any direction were excluded. And the mean framewise displacement (FD) was used to evaluate head motion and subjects with FD > 0.5 were excluded from our research. Finally, data were transformed using Fisher’s *z* to yield normally distributed data.

### Functional Network Construction

The functional networks comprised two vital components such as nodes and edges. As indicated in previous studies (Salvador et al., 2005; Lei et al., 2015; Xu et al., 2016), we also employed the AAL atlas (Supplementary Table 1) to divide a brain into 90 cortical and subcortical regions of interest (45 for each hemisphere), which were defined as 90 nodes in a brain network. Then, we extracted the average time course of each node and calculated the correlation coefficient between the two nodes. These functional connection matrices were defined as edges of a network, resulting in a 90 × 90 matrices. During the entire process of analysis, age, gender, and education level were used as covariates.

### Graph Theory Analysis

To ensure the same numbers of sides for each subject, we applied a range of sparse threshold (*S*) instead of a single threshold, and the criteria of the *S* section were in accordance with prior study (Mechelli et al., 2005) (1): the minimum of *S* was greater than 2log(*N*), and *N* is 90 in this study; (2) the maximum of *S* should guarantee the small-worldness σ > 1.1. So that the *S* ranged from 0.1 to 0.2 with the pitch being 0.01 here.

We investigated the global and nodal properties of the “small world” network in SNHL and HCs groups. The global characteristics (Wang et al., 2010) include: (1) small worldness; (2) cluster coefficient (Cp); (3) characteristic path length (Lp); (4) local efficiency (Eloc); and (5) global efficiency (Eglob). Node properties include: (1) degree centrality (measuring information communication ability in the functional network) (Bullmore and Sporns, 2009); (2) betweenness centrality (reflecting effects on information flow between other nodes) (Xu et al., 2019a); (3) nodal efficiency (representing nodes’ efficiency of parallel information transfer) (Rubinov and Sporns, 2010); (4) nodal clustering coefficient (evaluating the likelihood that neighborhoods of nodes are connected to each other) (van den Heuvel and Hulshoff Pol, 2010). The area under curve (AUC) was applied to facilitate the between-group comparison of network features (Bruno et al., 2012; Suo et al., 2015). Moreover, we did an interregional connectivity analysis among 90 nodes to find alterations following partial auditory deprivation. Two-sample *t*-test with multiple corrections was conducted in between-group analysis.

### Correlation Analysis Between Topologic Properties and Clinical Data

After extracting the group difference of topologic characteristics between SNHL and HCs, we computed Pearson’s correlation analysis to explore the potential relationships between functional data and clinical parameters. p < 0.05 was considered statistically significant.

## Results

### Demographics and Clinical Measurements

Detailed information about SNHL and HCs groups is summarized in Table 1. There was no significant difference in terms of gender, age, and education. The mean hearing thresholds of the left and the right ears in the SNHL group were much higher than HCs (*p* < 0.001) and the HCs group met the diagnosis of normal hearing (<25 dB). In a series of neuropsychological scales, we did not find significance in the MMSE test, while the SNHL subjects performed worse in SDMT (*p* = 0.001) and AVLT tests than HCs. During the process of five learning trials, SNHL group got higher scores in the first trial of AVLT (*p* = 0.001) and 5-min delayed recall (*p* = 0.024). There was a trend in the second trial of AVLT (*p* = 0.054) and 20-min delayed recall (*p* = 0.027).

### Characteristics of Small-World Network

#### Global Level

**FIGURE 1:**
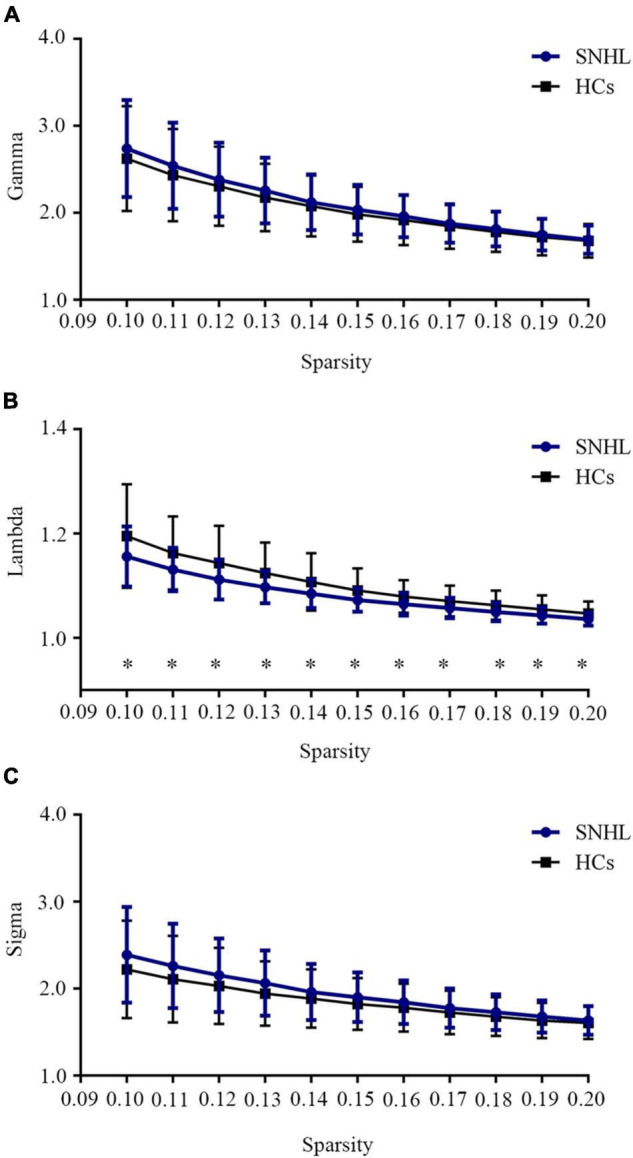
Small-world network properties of SNHL and HCs over a defined range of sparsity (0.10–0.20). **(A)** Comparison of brain network Gamma values between SNHL and HCs; **(B)** comparison of brain network Lambda values between SNHL and HCs; **(C)** comparison of brain network Sigma values between SNHL and HCs. Both groups had typical small-world features, as gamma > 1, lambda ≈ 1, sigma > 1. Values of lambda in SNHL group were significantly lower than HCs. SNHL, sudden sensorineural hearing loss; HCs, healthy controls. *p < 0.05.

**FIGURE 2:**
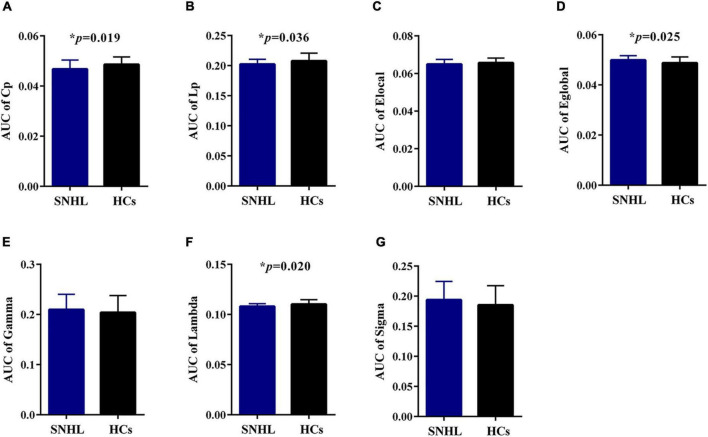
Graph theoretical measurements of whole brain network in SNHL group (blue) and HCs (black). **(A)** Compared with HCs, the SNHL group had significantly lower AUC of Cp; **(B)** SNHL group had significantly lower AUC of Lp; **(C)** there was no significant difference in AUC of local efficiency; **(D)** the SNHL group had significantly higher AUC of global efficiency than HCs; **(E)** there was no significant difference in AUC of gamma; **(F)** the SNHL group had significantly lower AUC of lambda; **(G)** there was no significant difference in AUC of sigma. SNHL, sudden sensorineural hearing loss; HCs, healthy controls; AUC, area under curve; Cp, cluster coefficient; Lp, characteristic path length. *p < 0.05.

As shown in Figure 1, SNHL and HCs groups both had typical features of the small-world network (Lei et al., 2015; Luo et al., 2015), as the values of gamma and sigma were larger than 1, and the values of lambda were close to 1 over the sparsity (0.1–0.2). In addition, after the two-sample *t*-test, we observed decreased values of lambda in every sparsity threshold in the SNHL group. Figure 2 shows the AUC of Cp, Lp, Elocal, Eglobal, gamma, lambda, and sigma. Compared with HCs, the SNHL displayed lower AUC of Cp (*p* = 0.019, Figure 2A), Lp (*p* = 0.036, Figure 2B) and lambda (*p* = 0.020, Figure 2F). However, the global efficiency increased in SNHL group (*p* = 0.025, Figure 2D). No significant difference was identified in Elocal (Figure 2C), gamma (Figure 2E), and sigma (Figure 2G).

#### Local Level

**FIGURE 3:**
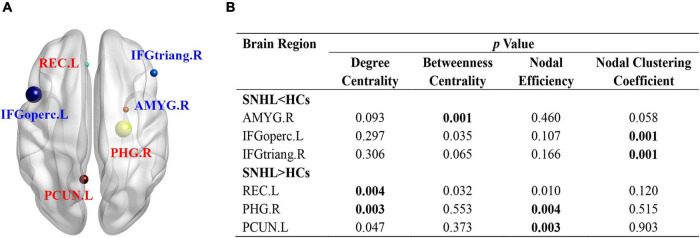
Nodal characteristics of SNHL group and HCs. **(A)** Shows the spatial distribution of significant nodes; **(B)** *p* values of nodal parameters, including degree centrality, betweenness centrality, nodal efficiency, and nodal clustering coefficient. Significant p was set at < 0.005. SNHL, sudden sensorineural hearing loss; HCs, healthy controls; L, left; R, right; ORBmid, orbital part of middle frontal gyrus; MFG, middle frontal gyrus; INS, insula; ROL, Rolandic operculum; ORBinf, orbital part of inferior frontal gyrus; TPOsup, temporal pole: superior temporal gyrus; DCG, median cingulate and paracingulate gyrus; HES, Heschl’s gyrus; STG, superior temporal gyrus; CAU, caudate nucleus; THA, thalamus; ITG, inferior temporal gyrus; HIP, hippocampus; FFG, fusiform gyrus; PreCG, precentral gyrus; PoCG, postcentral gyrus; PCL, paracentral lobule; SFGmed, superior frontal gyrus, medial; IOG, inferior occipital gyrus.

Supplementary Table 1 lists all names and abbreviations of 90 nodes in the AAL atlas. At an uncorrected threshold (*p* < 0.005), SNHL showed increased degree centrality in the left rectus gyrus (REC.L) and right parahippocampal gyrus (PHG.R), as well as increased nodal efficiency in PHG.R and left precuneus (PCUN.L). Compared with HCs, the right amygdala (AMYG.R) showed reduced betweenness centrality. The opercular part of the left inferior frontal gyrus (IFGoperc.L) and the triangular part of the right inferior frontal gyrus (IFGtriang.R) showed decreased nodal clustering coefficient in SNHL subjects (Figure 3).

### Interregional Connectivity

**FIGURE 4:**
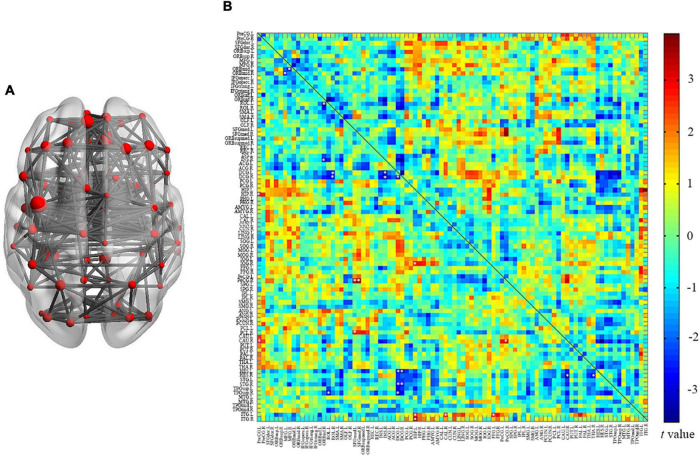
Between group analysis of internal cortical connectivity among 90 nodes from the AAL atlas. **(A)** Network construction of 90 nodes; **(B)** a matrix map of internal connectivity indicates *t* values of interactions. Significant p was set at < 0.005, uncorrected.

In this study, we reconstructed the network using 90 nodes (Figure 4A) and investigated the alterations of interregional connectivity following auditory deprivation (Figure 4B and Table 2) using a *p* < 0.005. In the SNHL group, the orbital part of the left middle frontal gyrus (ORBmid.L) showed a weakened connection with right middle frontal gyrus (MFG.R); the ORBimd.R showed decreased connections with MFG.L and right insula (INS.R). The left rolandic operculum (ROL.L) showed reduced connectivity with the orbital part of the right inferior frontal gyrus (ORBinf.R) and right temporal pole (superior temporal gyrus) (TPOsup.R), while ROL.R showed weakened connections with bilateral median cingulate and paracingulate gyrus (DCG.L and DCG.R). The connections between DCG.L and INS.R, DCG.R, left Heschl’s gyrus (HES.L), right superior temporal gyrus (STG.R), TPOsup.R, as well as the connections between DCG.R and INS.R, HES.L, STG.R were also decreased after hearing loss. The INS.R showed reduced connectivity with the right caudate nucleus (CAU.R) and thalamus (THA.R).

**TABLE 2:** Altered internal connectivity among 90 nodes following SNHL with tinnitus.

	Functional connectivity
**SNHL < HCs**	
1	ORBmid.L-MFG.R
2	ORBmid.R-MFG.L
3	ORBmid.R-INS.R
4	ROL.L-ORBinf.R
5	ROL.L-TPOsup.R
6	ROL.R-DCG.R
7	ROL.R-DCG.L
8	DCG.L-INS.R
9	DCG.L-DCG.R
10	DCG.L-HES.L
11	DCG.L-STG.R
12	DCG.L-TPOsup.R
13	DCG.R-INS.R
14	DCG.R-HES.L
15	DCG.R-STG.R
16	HES.R-CAU.R
17	HES.R-THA.R
**SNHL > HCs**	
1	ITG.L-HIP.L
2	ITG.L-CAU.R
3	ITG.L-FFG.L
4	ITG.R-HIP.L
5	CAU.R-PreCG.L
6	CAU.R-PoCG.R
7	PCL.R-SFGmed.L
8	PoCG.R-SFGmed.L
9	PoCG.R-SFGmed.R
10	IOG.R-HIP.L
11	HIP.R-PreCG.L

*SNHL, sensorineural hearing loss; HCs, healthy controls; L, left; R, right; ORBmid, orbital part of middle frontal gyrus; MFG, middle frontal gyrus; INS, insula; ROL, Rolandic operculum; ORBinf, orbital part of inferior frontal gyrus; TPOsup, temporal pole: superior temporal gyrus; DCG, median cingulate and paracingulate gyrus; HES, Heschl’s gyrus; STG, superior temporal gyrus; CAU, caudate nucleus; THA, thalamus; ITG, inferior temporal gyrus; HIP, hippocampus; FFG, fusiform gyrus; PreCG, precentral gyrus; PoCG, postcentral gyrus; PCL, paracentral lobule; SFGmed, superior frontal gyrus, medial; IOG, inferior occipital gyrus.*

Moreover, there were some increased connections in the SNHL group. The left inferior temporal gyrus (ITG.L) exhibited increased connectivity with the left hippocampus (HIP.L), CAU.R, and left fusiform gyrus (FFG.L). The ITG.R-HIP.L connectivity was increased too. The CAU.R showed enhanced connections with left the precentral gyrus (PreCG.L) and right postcentral gyrus (PoCG.R). The medial part of the left superior frontal gyrus (SFGmed.L) showed increased connectivity with the right paracentral lobule (PCL.R) and PoCG.R, while SFGmed.R showed increased connectivity with PoCG.R. Furthermore, the HIP.L showed enhanced connectivity with the right inferior occipital gyrus (IOG.R), while the HIP.R showed increased connection with PreCG.L.

### Relationships Between Topologic Properties and Clinical Data

**FIGURE 5:**
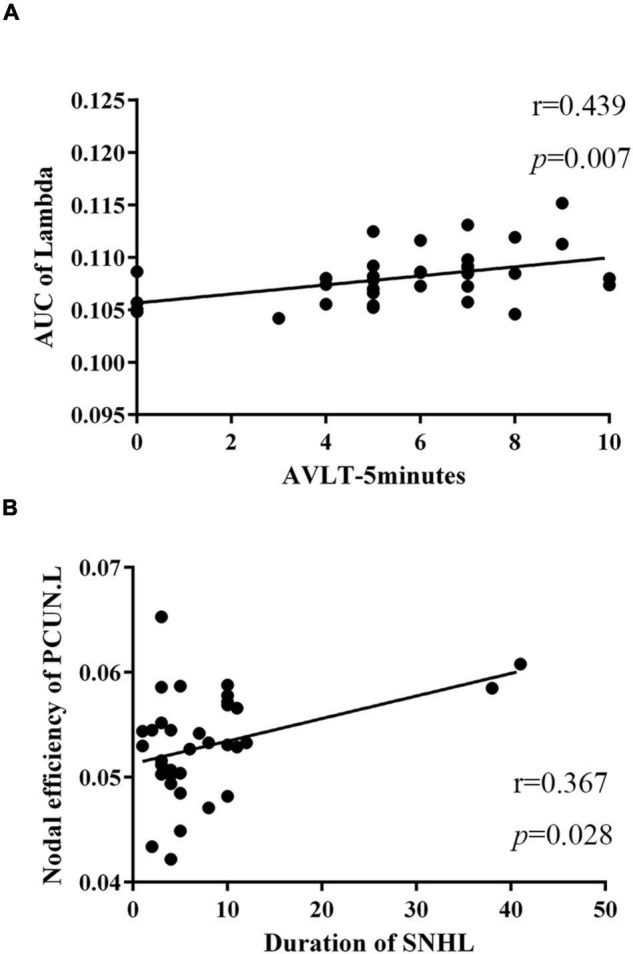
Relationships between topologic properties and clinical measurements in the SNHL group. **(A)** The AUC of lambda was positively correlated with scores of AVLT-5 min (*r* = 0.439, *p* = 0.007); **(B)** nodal efficiency of PCUN.L showed positive correlation with duration of SNHL (*r* = 0.367, *p* = 0.028). SNHL, sudden sensorineural hearing loss; AUC, area under curve; AVLT, auditory verbal learning test; PCUN, precuneus.

To further assess the relationships between topologic architectures and clinical variables, Pearson’s correlation analysis was conducted. In SNHL group, we found the AUC of lambda showed positive correlation with scores of AVLT-5 min (*r* = 0.439, *p* = 0.007, Figure 5A). And the nodal efficiency of PCUN.L was positively correlated with the duration of SNHL (*r* = 0.367, *p* = 0.028, Figure 5B).

## Discussion

This study examined the topologic properties of SNHL with tinnitus, providing a new perspective of functional connectome and cognition in auditory deprivation. Our findings revealed that compared with the HCs group, the SNHL group performed worse on cognitive scales, and had decreased clustering coefficient, path length, and AUC of lambda, but increased global efficiency. Significant alterations of nodal characteristics, including degree centrality, betweenness centrality, nodal efficiency, and nodal clustering coefficient, were observed in AMYG.R, IFGoperc.L, IFGtriang.R, REC.L, PHG.R, and PCUN.L. Besides, some internal connectivity among 90 nodes from AAL atlas was also changed following SNHL. We found positive correlations not only between the AUC of lambda and the performance of AVLT-5 min but also the nodal efficiency of PCUN.L and the duration of SNHL, indicating the potential mechanism of SNHL-induced cognitive impairments.

Our study applied graph theory analysis and small-world networks to better understand the neural plasticity after SNHL and tinnitus because the human brain is a large-scale and complex network with amounts of topologic features. Graph theory was widely used in mathematics and computer science in the past. Leonhard Euler (1736) firstly used graph theory to solve the “Königsberg Bridge Problem” in 1736, and then, it became popular in the biological neural networks, especially after the rise of “Human Connectome” project (Sporns et al., 2005). Ordinarily, a network was thought to have two types, as a regular network and a random network, while Watts and Strogatz (1998) defined a “small-world” network in 1998, which showed a high clustering coefficient and short path length, lying between regular and random network. In a “small-world” network, brain regions interact with adjacent areas to reduce metabolic and wiring costs (Bullmore and Sporns, 2009; Chen et al., 2013) and deviating from a “small-world” network indicates a disordered brain function.

At the global level, subjects with SNHL showed decreased clustering coefficient and path length in this study but increased Eglobal values. Kim et al. (2014) compared the graph-theory-based characteristics among prelingual deafness, postlingual deafness, and hearing subjects. They found that hearing subjects had a different distribution of clustering coefficient from prelingual deafness, but similar to postlingual deafness, as the clustering coefficient reflected the segregation of brain network (Bassett et al., 2008). Zhang et al. (2018) conducted a connectome-level analysis on unilateral hearing loss and failed to find any difference in global parameters, including Lp, Cp, Eglobal, and Elocal. In contrast, Xu et al. (2016) detected enhanced Cp and weakened Lp in unilateral SNHL. Chen et al. found that tinnitus patients have increased global efficiency, local efficiency, and cluster coefficient, indicating generally heightened connectivity of the network, which was consistent with the increased global efficiency of our study (Chen et al., 2020). These discrepancies might result from (1) various kinds of hearing loss, such as side, duration, and severity; (2) different nodes of network construction and our study chose 90 nodes from the AAL atlas; (3) differences in accompanied symptoms, such as tinnitus and vertigo. Auditory deprivation was known to induce network deficits, but SNHL patients here preserved part of their hearing ability so that increased global efficiency could be interpreted using compensatory reorganization theory. Additionally, lambda in all sparsity and the AUC of lambda in the SNHL group were lower than HCs. According to the previous study (Vecchio et al., 2014), lambda is referred to as characteristic path length, which is consistent of our Lp data. We also observed the positive correlation between the AUC of lambda and scores of AVLT-5 min, suggesting the relationship between lambda and cognition.

At the nodal level, 90 nodes denote 90 brain areas. The REC, which is located at the basal and inferior surface of the frontal lobe (Ballmaier et al., 2004), showed an increased degree centrality following SNHL in our study, indicating the involvement of frontal cortex in auditory deprivation disease. Degree centrality and betweenness centrality were used to evaluate the importance of nodes in the network. Degree centrality counts the number of neighbors of each node and betweenness centrality captures the ability to be a bridge for information flow (Mazrooyisebdani et al., 2018). It is reported that the function of REC is unclear and emerging evidence demonstrates that it might be involved in emotional processing and high cognition (Orrison, 2008). Bremner et al. found decreased orbitofrontal cortical metabolism (including REC) in depressed patients using PET in 1997 (Bremner et al., 1996) and a smaller volume of REC in 2002 (Bremner et al., 2002), suggesting its implication in depression. Accolla et al. (2016) used the posterior REC as a target for deep brain stimulation to treat resistant depression, considering its reciprocal projections to the medial prefrontal cortex and other areas in the limbic system (Kringelbach and Rolls, 2004). However, we observed enhanced degree centrality in the REC, which might result from cognitive overload and great efforts to listen after SNHL. It might be consistent with the increased nodal features of PCUN and PHG since these two regions are major nodes in the dorsal attention network and default mode network, respectively. Moreover, the nodal efficiency of PCUN showed a positive correlation with the duration of SNHL, as it consumed 35% more glucose than other cortex during high cognitive tasks (Li et al., 2021).

The IFGoperc and IFGtriang showed decreased nodal clustering coefficient in the SNHL group, which was partially in accordance with our previous findings in long-term deafness (Xu et al., 2019c), as the connections between the cerebellum and IFG were reduced. Bidelman et al. (2019) recently demonstrated subjects with hearing impairments had less efficient communication among widespread networks than normal hearing subjects and reversed directional IFG→auditory cortex connectivity using a speech-in-noise identification task. The above pointed out that different subregions of the frontal lobe might play different roles in brain function, and further research needs to address this specificity. Besides, we observed decrease in centrality in AMY and it is known as a core area in emotion. Further study could add the emotional effects of SNHL.

The internal connectivity among 90 nodes was widely changed following SNHL-induced parietal auditory deprivation, demonstrating that hearing loss is not only a peripheral neural system disease but also a central neural system disease, affecting more than the traditional auditory network. Therefore, we need to shed light on the role of the brain connectome and expand networks of interest in subsequent analysis.

There were some limitations in our study, as follows: (1) small sample size; (2) various duration of SNHL, future work needs to recruit more subjects and control confounding factors. Sub-group analysis could be conducted to deeply analyze the detailed information, such as the effects of different severities and durations on brain characteristics; (3) different frequencies, SNHL subjects might suffer from hearing loss at diverse frequencies, which could contribute to distinct influence; (3) choice of 90 nodes, considering the lack of criteria of standard templates, we chose AAL atlas to construct the network in this study, which omitted the cerebellum. Future studies had better include the cerebellum using an accepted template, while the establishment of this template is another research topic.

## Conclusion

This study found the connectome abnormalities from the global and nodal levels following SNHL, as well as the interregional connectivity. Some of the altered properties were significantly correlated with clinical measurements. Our research might disclose the potential importance of these imaging biomarkers and provide evidence for early treatment of SNHL-induced cognitive impairments.

## Data Availability Statement

The original contributions presented in the study are included in the article/Supplementary Material, further inquiries can be directed to the corresponding author/s.

## Ethics Statement

The studies involving human participants were reviewed and approved by the Research Ethics Committee of the Nanjing Medical University. The patients/participants provided their written informed consent to participate in this study.

## Author Contributions

J-CH and X-MX collected the MRI data, performed the analysis, and wrote the manuscript. Z-GX helped with the data analysis and discussion. J-JX and J-HH contributed to the data analysis. YW and YX designed the study and helped with revisions. All authors contributed to the article and approved the submitted version.

## Conflict of Interest

The authors declare that the research was conducted in the absence of any commercial or financial relationships that could be construed as a potential conflict of interest.

## Publisher’s Note

All claims expressed in this article are solely those of the authors and do not necessarily represent those of their affiliated organizations, or those of the publisher, the editors and the reviewers. Any product that may be evaluated in this article, or claim that may be made by its manufacturer, is not guaranteed or endorsed by the publisher.

## Footnotes

1
https://www.nitrc.org/projects/gretna

2
https://www.mathworks.com/
