# Supplementary material for: Aberrant Functional Network of Small-World in Sudden Sensorineural Hearing Loss With Tinnitus
Source: Front Neurosci. 2022 May 19;16:898902. doi: 10.3389/fnins.2022.898902 (PMC9160300; doi:10.3389/fnins.2022.898902)
Supplement: Supplementary file 1 [file Table_1.DOCX]

Supplementary Material

# Supplementary Table

Table S1. Detailed information of ninety nodes from AAL atlas.

| Number | Abbreviation | Brain region |
| --- | --- | --- |
| 1 | PreCG.L | Left precentral gyrus |
| 2 | PreCG.R | Right precentral gyrus |
| 3 | SFGdor.L | Left superior frontal gyrus, dorsolateral |
| 4 | SFGdor.R | Right superior frontal gyrus, dorsolateral |
| 5 | ORBsup.L | Left superior frontal gyrus, orbital part |
| 6 | ORBsup.R | Right superior frontal gyrus, orbital part |
| 7 | MFG.L | Left middle frontal gyrus |
| 8 | MFG.R | Right middle frontal gyrus |
| 9 | ORBmid.L | Left middle frontal gyrus, orbital part |
| 10 | ORBmid.R | Right middle frontal gyrus, orbital part |
| 11 | IFGoperc.L | Left inferior frontal gyrus, opercular part |
| 12 | IFGoperc.R | Right inferior frontal gyrus, opercular part |
| 13 | IFGtriang.L | Left inferior frontal gyrus, triangular part |
| 14 | IFGtriang.R | Right inferior frontal gyrus, triangular part |
| 15 | ORBinf.L | Left inferior frontal gyrus, orbital part |
| 16 | ORBinf.R | Right inferior frontal gyrus, orbital part |
| 17 | ROL.L | Left Rolandic operculum |
| 18 | ROL.R | Right Rolandic operculum |
| 19 | SMA.L | Left supplementary motor area |
| 20 | SMA.R | Right supplementary motor area |
| 21 | OLF.L | Left olfactory cortex |
| 22 | OLF.R | Right olfactory cortex |
| 23 | SFGmed.L | Left superior frontal gyrus, medial |
| 24 | SFGmed.R | Right superior frontal gyrus, medial |
| 25 | ORBsupmed.L | Left superior frontal gyrus, medial orbital |
| 26 | ORBsupmed.R | Right superior frontal gyrus, medial orbital |
| 27 | REC.L | Left rectus gyrus |
| 28 | REC.R | Right rectus gyrus |
| 29 | INS.L | Left insula |
| 30 | INS.R | Right insula |
| 31 | ACG.L | Left anterior cingulate and paracingulate gyrus |
| 32 | ACG.R | Right anterior cingulate and paracingulate gyrus |
| 33 | DCG.L | Left median cingulate and paracingulate gyrus |
| 34 | DCG.R | Right median cingulate and paracingulate gyrus |
| 35 | PCG.L | Left posterior cingulate gyrus |
| 36 | PCG.R | Right posterior cingulate gyrus |
| 37 | HIP.L | Left hippocampus |
| 38 | HIP.R | Right Hippocampus |
| 39 | PHG.L | Left parahippocampal gyrus |
| 40 | PHG.R | Right parahippocampal gyrus |
| 41 | AMYG.L | Left amygdala |
| 42 | AMYG.R | Right amygdala |
| 43 | CAL.L | Left calcarine fissure and surrounding cortex |
| 44 | CAL.R | Right calcarine fissure and surrounding cortex |
| 45 | CUN.L | Left cuneus |
| 46 | CUN.R | Right Cuneus |
| 47 | LING.L | Left lingual gyrus |
| 48 | LING.R | Right lingual gyrus |
| 49 | SOG.L | Left superior occipital gyrus |
| 50 | SOG.R | Right superior occipital gyrus |
| 51 | MOG.L | Left middle occipital gyrus |
| 52 | MOG.R | Right middle occipital gyrus |
| 53 | IOG.L | Left inferior occipital gyrus |
| 54 | IOG.R | Right inferior occipital gyrus |
| 55 | FFG.L | Left fusiform gyrus |
| 56 | FFG.R | Right fusiform gyrus |
| 57 | PoCG.L | Left postcentral gyrus |
| 58 | PoCG.R | Right postcentral gyrus |
| 59 | SPG.L | Left superior parietal gyrus |
| 60 | SPG.R | Right superior parietal gyrus |
| 61 | IPL.L | Left inferior parietal lobule |
| 62 | IPL.R | Right inferior parietal lobule |
| 63 | SMG.L | Left supramarginal gyrus |
| 64 | SMG.R | Right supramarginal gyrus |
| 65 | ANG.L | Left angular gyrus |
| 66 | ANG.R | Right angular gyrus |
| 67 | PCUN.L | Left precuneus |
| 68 | PCUN.R | Right precuneus |
| 69 | PCL.L | Left paracentral lobule |
| 70 | PCL.R | Right paracentral lobule |
| 71 | CAU.L | Left caudate nucleus |
| 72 | CAU.R | Right caudate nucleus |
| 73 | PUT.L | Left putamen |
| 74 | PUT.R | Right putamen |
| 75 | PAL.L | Left pallidum |
| 76 | PAL.R | Right pallidum |
| 77 | THA.L | Left thalamus |
| 78 | THA.R | Right thalamus |
| 79 | HES.L | Left Heschl’s gyrus |
| 80 | HES.R | Right Heschl’s gyrus |
| 81 | STG.L | Left superior temporal gyrus |
| 82 | STG.R | Right superior temporal gyrus |
| 83 | TPOsup.L | Left temporal pole: superior temporal gyrus |
| 84 | TPOsup.R | Right temporal pole: superior temporal gyrus |
| 85 | MTG.L | Left middle temporal gyrus |
| 86 | MTG.R | Right middle temporal gyrus |
| 87 | TPOmid.L | Left temporal pole: middle temporal gyrus |
| 88 | TPOmid.R | Right temporal pole: middle temporal gyrus |
| 89 | ITG.L | Left inferior temporal gyrus |
| 90 | ITG.R | Right inferior temporal gyrus |
